# ASO Author Reflections: Stage-Adjusted Reduced Follow-Up of Melanoma Patients is Justified and Cost Effective, Until Biomarkers to Predict Prognosis Have Been Identified

**DOI:** 10.1245/s10434-019-07611-5

**Published:** 2019-09-03

**Authors:** Anne Brecht Francken, Josette E. H. M. Hoekstra-Weebers, Eric Deckers, Harald J. Hoekstra

**Affiliations:** 1 Department of Surgical Oncology, Isala Clinics, Zwolle, The Netherlands; 2 Wenckebach Institute, University of Groningen, University Medical Center Groningen, Groningen, The Netherlands; 3 Department of Surgical Oncology, University of Groningen, University Medical Center Groningen, Groningen, The Netherlands

## Past

The incredible rise in melanoma health care costs urgently demands a reduction in these costs where appropriate.^1^ Nevertheless, cancer patients demand frequent and close follow-up out of fear of recurrence. Historically, melanoma patients have been followed regularly, with limited therapeutic options in case of disease progression.^2^ In addition, survival benefit as a result of follow-up has never been demonstrated.^3^ There is a lack of international consensus regarding the follow-up frequency of melanoma patients,^4^ and evidence regarding the optimal follow-up frequency of these patients with respect to disease-free and overall survival, patients’ quality of life (QoL), and costs is highly needed.

## Present

The current randomized controlled MELFO study compared two groups of stage Ib–IIc melanoma patients, 3 years after diagnosis.^5^ The first group received follow-up as advised in the guideline, while the second group received a stage-adjusted, less frequent follow-up schedule. Patients’ QoL, anxiety, satisfaction regarding follow-up, and disease-free and overall survival were comparable, but a 39% cost reduction was found in those who were less frequently followed-up. A reduced and stage-adjusted follow-up schedule could be a step forward in better distribution of resources, such as finances, time, and manpower.

## Future

Several questions need to be answered in the future to determine the optimal, safe, (cost)-effective follow-up that will benefit all melanoma patients.^3^ Apart from recurrence detection, mental support and patient education are important after-care goals for melanoma patients with any stage of disease.^6^^,^^7^ Now that several effective therapeutic adjuvant systemic treatment options with drug targeting and/or immunotherapy have become available, follow-up has become even more complex.^8^ What is the best strategy to improve OS in stage IB–II melanoma? Adjuvant therapy of high-risk stage II patients or treatment at the time of recurrence? How to select patients who will benefit from adjuvant treatment while sparing those who are unlikely to benefit from toxic effects? If melanoma biomarkers could be identified that can better predict the potential to metastasize than the current prognostic factors do, a personalized follow-up, including emotional support and patient education, could be delivered even more (cost) effectively. Currently, stage-adjusted follow-up is the best personalized follow-up approach for stage IB–II melanoma.
